# Recruiting participants to walking intervention studies: a systematic review

**DOI:** 10.1186/1479-5868-8-137

**Published:** 2011-12-15

**Authors:** Charlie E Foster, Graham Brennan, Anne Matthews, Chloe McAdam, Claire Fitzsimons, Nanette Mutrie

**Affiliations:** 1 Department of Public Health, University of Oxford, UK; 2 Scottish Physical Activity Research Collaboration (SPARColl) & School of Psychological Sciences and Health, University of Strathclyde, Glasgow, UK

**Keywords:** Recruitment, walking, physical activity, health promotion

## Abstract

**Purpose:**

Most researchers who are conducting physical activity trials face difficulties in recruiting participants who are representative of the population or from specific population groups. Participants who are often the hardest to recruit are often those who stand to benefit most (the least active, from ethnic and other minority groups, from neighbourhoods with high levels of deprivation, or have poor health). The aim of our study was to conduct a systematic review of published literature of walking interventions, in order to identify the impact, characteristics, and differential effects of recruitment strategies among particular population groups.

**Methods:**

We conducted standard searches for studies from four sources, (i) electronic literature databases and websites, (ii) grey literature from internet sources, (iii) contact with experts to identify additional "grey" and other literature, and (iv) snowballing from reference lists of retrieved articles. Included studies were randomised controlled trials, controlled before-and-after experimental or observational qualitative studies, examining the effects of an intervention to encourage people to walk independently or in a group setting, and detailing methods of recruitment.

**Results:**

Forty seven studies met the inclusion criteria. The overall quality of the descriptions of recruitment in the studies was poor with little detail reported on who undertook recruitment, or how long was spent planning/preparing and implementing the recruitment phase. Recruitment was conducted at locations that either matched where the intervention was delivered, or where the potential participants were asked to attend for the screening and signing up process. We identified a lack of conceptual clarity about the recruitment process and no standard metric to evaluate the effectiveness of recruitment.

**Conclusion:**

Recruitment concepts, methods, and reporting in walking intervention trials are poorly developed, adding to other limitations in the literature, such as limited generalisability. The lack of understanding of optimal and equitable recruitment strategies evident from this review limits the impact of interventions to promote walking to particular social groups. To improve the delivery of walking interventions to groups which can benefit most, specific attention to developing and evaluating targeted recruitment approaches is recommended.

## Introduction

It is over a decade since Professors Jerry Morris and Adrienne Hardman described walking as the 'nearest activity to perfect exercise' (Hardman & Morris, p328, 1997) [1]. The epidemiological research underpinning their statement has rapidly increased, so that the promotion of walking is now a central pillar in many international physical activity strategies and national plans, e.g. 2010 Toronto Charter for Physical Activity [2]. Regular walking, independent of other physical activity, can reduce the risk of overall mortality, of cardiovascular disease (CVD) and improve risk factors for CVD, including diastolic blood pressure and lipid profiles [3-5]. Regular walking is associated with a reduction in body mass index and body weight, with reduced risk of type 2 diabetes [6] and is suggested to improve self esteem, relieve symptoms of depression and anxiety, and improve mood [7,8]. From a public health perspective, enabling an increase in overall population levels of physical activity through walking will produce an effective reduction in risk of all cause mortality [9].

A systematic review of the effectiveness of walking interventions found evidence for a range of approaches [10]. These included brief advice to individuals, remote support to individuals, group-based approaches, active travel and community level approaches. Recent reviews have provided evidence to support environmental and school based travel interventions [10-12]. Despite the evidence for the benefits of walking for health, population rates of walking and overall physical activity remain low and below recommended levels [13-15]. Population surveys report that walking behaviour is socially patterned by gender, age, socio-economic status (SES) and by the purpose of walking i.e. for leisure or transport. For example, in the UK long brisk paced walks are more common among affluent groups, whereas walking for transport is more common among less affluent groups [14,16].

One criticism of the evidence base for walking interventions is a failure to recruit specific groups of the population and further studies are needed to broaden the reach of walking interventions [10-12]. Intervention reach, or recruiting specific population sub-groups, is only partially reflected in public health and clinical research. For example the RE-AIM framework is designed to guide the implementation of behaviour change interventions [17]. It recommends assessing both an intervention's effectiveness and ability to reach a targeted group. Similarly, recent CONSORT (2010) guidelines [18] recommend clearly displaying the flow of participants throughout a study. Despite identifying recruitment as part of their framework, the guidelines do not define the actions needed to identify and recruit potential populations of participants. There is an absence of conceptual frameworks for recruitment to intervention studies and also a lack of procedural models and systems for recruitment. There is a need to identify what factors are effective in engaging participation at the recruitment phase [19-21].

Research examining recruitment practice has focused on drug or medical interventions rather than public health interventions [22]. Little is known about recruitment to physical activity interventions. A Cochrane review identified three stages of recruitment (invitation, screening, intervention starting) for potential participants into physical activity randomised control trials (RCTs). The authors noted a considerable loss of participants across each stage limiting the effectiveness of interventions [23]. The CONSORT (2010) guidelines, suggest that studies report the number of eligible participants prior to randomisation but do not insist on the need to report the original overall number of responders invited to participate (prior to eligibility) [18].

Clearly the effectiveness of a walking programme is limited by not only its efficacy of dose (how well the intervention works on its participants) but also by its recruitment (maximising the numbers who will participate and receive the intervention dose). In response to frequent research calls to evaluate effective approaches to the recruitment of individuals to walking studies, the Scottish Physical Activity Research Collaboration http://www.sparcoll.org.uk undertook a series of studies to examine recruitment strategies for research and community based programmes of walking promotion. We defined recruitment for such walking studies or programmes as the process of inviting participation to a formal activity including the invitation, informing and facilitation of interested parties to take part in an organised study, activity or event. This paper reports the results of a systematic review to examine the reported recruitment procedures of walking studies, in order to identify the characteristics of effective recruitment, and the impact and differential effects of recruitment strategies among particular population groups.

## Method

### Identification of studies

We used The Quality of Reporting of Meta-analysis statement (QUOROM) to provide the structure for our review [24]. We identified four possible sources of potential studies, (i) electronic literature databases and websites, (ii) grey literature from internet sources, (iii) contact with experts to identify additional "grey" and other literature, and (iv) snowballing from reference lists of retrieved articles. In the first stage of the literature search, titles and abstracts of identified articles were checked for relevance. In the second stage, full-text articles were retrieved and considered for inclusion. In the final stage, the reference lists of retrieved full-text articles were searched and additional articles known to the authors were assessed for possible inclusion. We conducted a systematic search of electronic databases including OVID MEDLINE, EMBASE, PsychINFO, PubMed, Scopus, SIGLE and SPORTDiscus. We searched a number of web based databases including National Institute of Health and Clinical Excellence (NICE), Effective Public Health Project (EPHP Hamilton), Health Evidence Canada, and the Evidence for Policy and Practice Information and Co-ordinating Centre (EPPI)). We conducted searches of internet sites of key international walking promotion agencies including Walk England, the Centers for Disease Control and Prevention (CDC) and the World Health Organisation (WHO).

Studies published from the end of 2000 up to and including the search date (05/2009) were considered for inclusion. Individualized search strategies for the different databases included combinations of the following key words: (walk*) AND (recruit* OR participat* OR market*). Articles published or accepted for publication in refereed journals were considered for the review. Articles reported in UK grey and web based literature including any evidence of types of recruitment approaches and strategies, any evidence of effectiveness, economic costs, and evidence of any differential response to recruitment approaches were also considered in the review. Conference proceedings and abstracts were included if further searching of the databases or contact with the author was able to retrieve a full article from the study presented in the original piece of literature. We sent emails to international experts, identified in a previous systematic review on walking promotion [10].

### Criteria for study inclusion/exclusion

Titles, abstracts and reports were independently assessed (by AM, CF and GB) for inclusion. Studies were considered to be eligible for inclusion according to the following criteria: (i) participants were of any age and were not trained athletes or sports students, (ii) studies of any type including randomised controlled trials, controlled before-and-after experimental or observational studies, (iii) studies that examined the effects of an intervention to encourage people to walk independently or in a group setting, (iv) interventions of any kind and in any field, whether targeted on individuals, communities, settings, groups or whole populations, (v) details of methods of recruitment were reported or were retrievable through correspondence with the authors, (vi) qualitative studies that examined the experiences of the participants during recruitment and which aimed to assess the effectiveness of the recruitment methods used, and (vii) studies published in English.

Included studies were categorised by study design using standardised criteria for quantitative experimental or observational studies (e.g. RCT, non-Randomised Control Trials (NRCT), before-and-after, cross-sectional), or qualitative studies (e.g. focus groups) [25].

### Criteria for assessment of study quality in relation to recruitment

Two authors (GB and CF) independently assessed the quality of the studies in relation to recruitment description that met the inclusion criteria. The criteria for assessing the recruitment reporting quality of each study were adapted from Jadad (1998) [26], and in consultation with experts. A formal quality score for each study was completed on a 5-point scale by assigning a value of 0 (absent or inadequately described) or 1 (explicitly described and present) to each of the following questions listed: (i) did the study report where the population was recruited? (ii) did the study report who conducted the recruitment? (iii) did the study report the time spent planning/preparing the recruitment? (iv) did the study report the time spent conducting the recruitment? (v) did the study target a specific population? Studies that scored 4-5 were considered as high quality studies while studies that scored 1-3 were considered low quality.

### Criteria for assessing efficiency and effectiveness

Where possible we calculated recruitment rates and efficiency ratios for each study, based on a previous systematic review of interventions to promote physical activity [23]. We defined four terms, (i) "pool"-the total number of potential participants who could be eligible for study, (ii) "invited"-the total number of potential participants invited to participate in the study, (iii) "responded"-the total number of potential participants who responded to the invitation, (iv) "started"-the number of participants who were assessed as eligible to participate and began the programme. If data were reported we calculated ratios for each stage, e.g. started/pool-by dividing the number of participants who started into the study by the total reported in the pool, and expressed as proportions. If possible we attempted to calculate a weekly rate of recruitment for those studies on the number of weeks/months spent recruiting per participant.

## Results

### Study Characteristics

**Figure 1:**
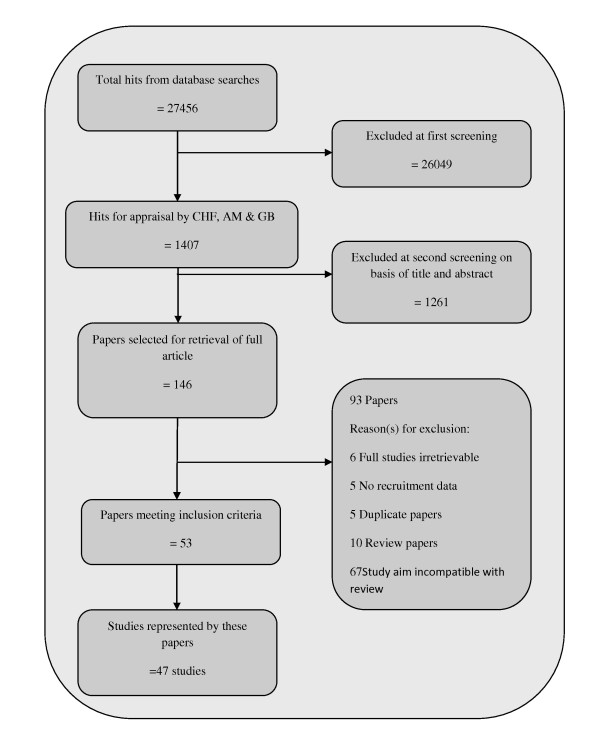
**Flow diagram of study selection**.

Fifty three papers representing 47 studies met our inclusion criteria. Duplicate studies were excluded and the journal article reporting the most recruitment data was analysed. The flow of studies through the review process is reported in Figure 1. Characteristics of included studies are presented in Table 1, ranked by quality score. Each included paper is referenced in the results and discussion sections in superscript, using their Study Number presented in Table 1. Full references for included papers are listed in additional file 1 and are presented in this paper in superscript form. Studies were located in the USA (24) [27-50], Australia (11) [51-61], UK (7) [62-68], Canada (3) [69-71], and one each from New Zealand [72] and Belgium [73]. Nearly all the studies were quantitative experimental studies in design, with twenty six randomised controlled trials, [4,27,28,32-34,36-38,42,43,46,47,49,52,54,56,58,62-67,70] two studies reporting methods only [28,35], three non-randomised controlled trials [31,41,73] and seventeen before-and-after studies [27,29,30,39,40,44,45,48,50,51,53,55,59-61,68,71] (two reporting methods only) [27,30]. We found only two qualitative studies reporting on recruitment approaches [57,69], with one paper reporting qualitative data as part of an RCT study [64]. No studies were located from grey literature sources.

**Table 1:** Characteristics of included studies

Study Number, Author and Pub. Year	Country	Study Type	Study aim	Target Population	Quality Metric Score
1. Watson et al, 2005	Australia	Before-and-after study	Evaluate the effect of pram walking groups on self-reported PA, mental health and social indicators.	Post-natal mothers	5
2. Banks-Wallace et al, 2004	USA	Before-and-after study Methods paper	Examine the effect of pre-intervention meetings as a strategy for recruitment of African American women to a walking programme.	African American women in a local community (Minority group)	4
3. Kolt et al, 2006	New Zealand	RCT	To investigate the effectiveness of a telephone-based counselling intervention aimed to increase physical activity in sedentary older adults.	Older sedentary adults (> 65)	4
4. Nguyen et al, 2002	Canada	Qualitative	To evaluate the experience of delivering a walking club (qualitative method)	General community	4
5. Prestwich et al, 2010	UK	RCT	To test the effect of implementation intentions and text messages on the promotion of brisk walking.	University students	4
6. Rowland et al, 2004	USA	RCT Methods paper	To report on the recruitment of sedentary adults to the SHAPE programme	Sedentary older adults	4
7. Sherman et al, 2006	USA	Before-and-after study	Effect of a brief primary care based walking intervention in rural women	Rural women	4
8. Wilbur et al, 2006	USA	Before-and-after study Methods paper	To identify strategies successful in the recruitment of African American women to a home-based walking programme and to examine the factors that contribute to attrition, eligibility, and ineligibility during the recruitment screening protocol.	African American Women	4
9. Baker et al, 2008b	UK	RCT	Effectiveness of pedometer based community walking intervention on PA and health	Community members in areas of high deprivation (> 15% SIMD)	3
10. Brownson et al 2005	USA	NRCT	To evaluate the impact of community based walking approaches	Rural community members	3
11. Cox et al, 2008	Australia	RCT	Examine the effects of exercise mode and a behavioural intervention on short and long-term retention and adherence.	Previously sedentary older women	3
12. Dinger et al, 2007	USA	RCT	Compare the effectiveness of two email delivered, pedometer based interventions designed to increase walking and TTM constructs among insufficiently active women.	Insufficiently active women (University staff and local community members)	3
13. Dubbert et al, 2002	USA	RCT	Effect of nurse counselling on walking for exercise in elderly patients (10 months study)	Elderly primary care patients	3
14. Dubbert et al, 2008	USA	RCT	To evaluate the effects of counselling linked with PHC visits on walking and strength exercise in aging veterans	Elderly veterans	3
15. Gilson et al, 2008	UK	RCT, Qualitative	To compare two walking interventions and measure their effect on daily step counts in a work-place environment	Work-place employees	3
16. Jancey et al, 2008	Australia	Before-and-after study	To mobilise older adults into a neighbourhood-based walking programme	Older adults	3
17. Lamb et al, 2002	UK	RCT	To compare lead walks vs. advice only on PA (walking)	Middle aged adults	3
18. Lee et al, 1997	USA	RCT Methods paper	To compare the efficacy of a mail versus phone based behavioural intervention to promote walking for US adults	Sedentary ethnic minority women	3
19. Matthews et al, 2007	USA	RCT	To evaluate the effects of a 12-week home-based walking intervention among breast cancer survivors	Breast cancer survivors	3
20. Merom et al, 2007	Australia	RCT	Efficacy of pedometers to act as a motivational tool in place of face to face contact as part of a self-help package to increase PA through walking.	Inactive adults	3
21. Ornes and Ransdell, 2007	USA	RCT	To evaluate the impact of a web-based intervention for women	Women	3
22. Richardson et al, 2007	USA	RCT	To compare the effects of structured and lifestyle goals in an internet-mediated walking programme for adults with type 2 diabetes	Adults with type 2 diabetes	3
23. Rosenberg et al, 2009	USA	Before-and-after study	Feasibility and acceptability of a novel multilevel walking intervention for older adults in a continuing care retirement community (CCRC).	Older adults	3
24. Whitt-Glover et al, 2008	USA	Before-and-after study	Feasibility and acceptability of implementing a physical activity program for sedentary black adults in churches. (Information sessions and lead walks)	Black adult, church attendees	3
25. Arbour & Ginis, 2009	Canada	RCT	Evaluate the effectiveness of implementation intentions on walking behaviour	Women in the workplace	2
26. Culos-Reed et al, 2008	Canada	Before-and-after study	To assess the feasibility and health benefits of a mall walking programme.	NS	2
27. Currie and Develin, 2001	Australia	Before-and-after study	To evaluate the impact of a community based pram walking programme-organised pram walks.	Mothers and young children	2
28. Darker et al, 2010	UK	RCT	To examine whether altering perceived behavioural control (PBC) affects walking (6/7 weeks).	NS	2
29. De Cocker et al 2007	Belgium	NRCT	Describe the effectiveness of the '10,000 steps Ghent' project.	'General population' adults in a local community	2
30. Dinger et al, 2005	USA	NRCT	Examine the impact of a 6 week minimal contact intervention on walking behaviour, TTM and self efficacy among women.	Female employees or spouses of university employees	2
31. Engel and Lindner, 2006	Australia	RCT	To evaluate the effect of a pedometer intervention on adults with type 2 diabetes	Adults with type 2 diabetes	2
32. Foreman et al, 2001	Australia	Qualitative	To increase the community's participation in physical activity through group walking	Community members	2
33. Humpel et al, 2004	Australia	RCT	Examine the effectiveness of self-help print materials and phone counselling in a study aimed specifically at promoting walking for specific purposes	Over 40 year old community members	2
34. Nies et al, 2006	USA	RCT	To increase walking activity in sedentary women (Video education, brief telephone calls without counselling, brief telephone calls with counselling)	European American and African America women.	2
35. Purath et al, 2004	USA	RCT	To determine if a brief, tailored counselling intervention is effective for increasing physical activity in sedentary women, in the workplace	Women in the workplace	2
36. Shaw et al, 2007	Australia	Before-and-after study	To evaluate a workplace pedometer intervention	Men and women in the workplace	2
37. Sidman et al, 2004	USA	Before-and-after study	Promote physical activity through walking	Sedentary women	2
38. Thomas and Williams, 2006	Australia	Before-and-after study	Increase activity through wearing a pedometer and encouraging participants to aim for 10,000 steps per day.	Workplace staff (Excluding hospital and community services staff)	2
39. Tudor-Locke et al, 2002	USA	Before-and-after study	Feasibility study of a community walking intervention	Sedentary diabetes sufferers	2
40. Baker et al, 2008a	UK	RCT	Examine the effectiveness of pedometers to motivate walking.	NS	1
41. Hultquist et al, 2005	USA	RCT	To compare the impact of two walking promotion messages	NS	1
42. Lomabrd et al, 1995	USA	RCT	To evaluate the effects of low v high prompting for walking	NS	1
43. DNSWH, 2002	Australia	Before-and-after study	To evaluate the impact of park modification, promotion of park use and establishment of walking groups on physical activity (including walking)	NS	1
44. Rovniak, 2005	USA	Before-and-after study	Examine the extent to which theoretical fidelity influenced the effectiveness of two walking programmes based on SCT.	NS	1
45. Rowley et al, 2007	UK	Before-and-after study	To examine the development of two walking programmes by a health visiting team to encourage undertaking of more exercise.	Parents and children	1
46. Talbot et al, 2003	USA	RCT	To evaluate the effects of a home based walking programme with arthritis self-management education	Older adults	1
47. Wyatt et al, 2004	USA	Before-and-after study	Increasing lifestyle physical activity (i.e. walking) for weight gain prevention	State wide residents of the community	1

### Overview of study quality in relation to recruitment

Eight studies were classified as "high" quality [27-30,51,62,69,72] and the remaining thirty nine classified as "low" quality in relation to recruitment description (Table 2-Assessment of study quality). Forty five studies reported a setting where the recruitment of participants took place [27-49,51-67,69-73] but only twenty two reported who conducted the recruitment [27-31,33,35-40,45,51-54,62,64,65,69,72]. Eleven studies reported the time spent conducting their recruitment [27-30,32,51,62,63,66,70,72] three studies reported the time spent planning/preparing recruitment [34,51,69]. Forty studies reported a target population [27-45,48,50-60,62-65,68-70,72,73].

**Table 2:** Assessment of study quality

Study Author (Year)	Did the study report where the population was recruited?	Did the study report who conducted the recruitment?	Did the study report the time spent planning/preparing the recruitment?	Did the study report the time spent conducting the recruitment?	Did the study target a specific population?	Quality Metric score
l. Watson et al, 2005	Yes	Yes	Yes	Yes	Yes	5
2. Banks-Wallace et al, 2004	Yes	Yes	No	Yes	Yes	4
3. Kolt et al, 2006	Yes	Yes	No	Yes	Yes	4
4. Nguyen et al, 2002	Yes	Yes	Yes	No	Yes	4
5. Prestwich et al, 2010	Yes	Yes	No	Yes	Yes	4
6. Rowland et al, 2004	Yes	Yes	No	Yes	Yes	4
7. Sherman et al, 2006	Yes	Yes	No	Yes	Yes	4
8. Wilbur et al, 2006	Yes	Yes	No	Yes	Yes	4
9. Baker et al, 2008b	Yes	No	No	Yes	Yes	3
10. Brownson et al 2005	Yes	Yes	No	No	Yes	3
11. Cox et al, 2008	Yes	Yes	No	No	Yes	3
12. Dinger et al, 2007	Yes	No	No	Yes	Yes	3
13. Dubbert et al, 2002	Yes	Yes	No	No	Yes	3
14. Dubbert et al, 2008	Yes	No	Yes	No	Yes	3
15. Gilson et al, 2008	Yes	Yes	No	No	Yes	3
16. Jancey et al, 2008	Yes	Yes	No	No	Yes	3
17. Lamb et al, 2002	Yes	Yes	No	No	Yes	3
18. Lee et al, 1997	Yes	Yes	No	No	Yes	3
19. Matthews et al, 2007	Yes	Yes	No	No	Yes	3
20. Merom et al, 2007	Yes	Yes	No	No	Yes	3
21. Ornes and Ransdell, 2007	Yes	Yes	No	No	Yes	3
22. Richardson et al, 2007	Yes	Yes	No	No	Yes	3
23. Rosenberg et al, 2009	Yes	Yes	No	No	Yes	3
24. Whitt-Glover et al, 2008	Yes	Yes	No	No	Yes	3
25. Arbour & Ginis, 2009	Yes	No	No	No	Yes	2
26. Culos-Reed et al, 2008	Yes	No	No	Yes	No	2
27. Currie and Develin, 2001	Yes	No	No	No	Yes	2
28. Darker et al, 2010	Yes	No	No	Yes	No	2
29. De Cocker et al 2007	Yes	No	No	No	Yes	2
30. Dinger et al, 2005	Yes	No	No	No	Yes	2
31. Engel and Lindner, 2006	Yes	No	No	No	Yes	2
32. Humpel et al, 2004	Yes	No	No	No	Yes	2
33. Nies et al, 2006	Yes	No	No	No	Yes	2
34. Purath et al, 2004	Yes	No	No	No	Yes	2
35. Shaw et al, 2007	Yes	No	No	No	Yes	2
36. Sidman et al, 2004	Yes	No	No	No	Yes	2
37. Thomas and Williams, 2006	Yes	No	No	No	Yes	2
38. Tudor-Locke et al, 2002	Yes	No	No	No	Yes	2
39. Foreman et al, 2001	No	Yes	No	No	Yes	2
40. Baker et al, 2008a	Yes	No	No	No	No	1
41. Hultquist et al, 2005	Yes	No	No	No	No	1
42. Lomabrd et al, 1995	Yes	No	No	No	No	1
43. DNSWH, 2002	Yes	No	No	No	No	1
44. Rovniak, 2005	Yes	No	No	No	No	1
45. Rowley et al, 2007	No	No	No	No	Yes	1
46. Talbot et al, 2003	Yes	No	No	No	No	1
47. Wyatt et al, 2004	No	No	No	No	Yes	1
Totals	45 Yes 2 No	22 Yes 25 No	3 Yes 44 No	11 Yes 36 No	40 Yes 7 No	

### Characteristics of the participants

Thirty seven studies reported participant ages [28-30,32-47,49,51-54,56,58,59,62-67,70-73] with a mean age of 50.6 years, (SD ± 8.1 years), and a range of 18 to 92 years (Table 3-Characteristics of participants). Sixteen out of forty two studies that reported gender data focused on recruiting female only participants [27,29,30,32,35-37,41-44,46,51,52,55,68,70], with one study recruiting men only [34]. From the remaining twenty five studies that did not recruit sex specific groups, 70% (SD ± 20.8) of participants were female. Twenty two studies reported data on nationality and ethnicity, of which seventeen reported descriptive statistics for ethnicity or race [27-38,40-42,46,49,51,54,58,68,70,71]. Three studies reported targeting one specific ethnic group, African-Americans [27,30,40]. Of the remaining studies, twelve reported other ethnicity data; 87% of these participants were white Caucasian [28,31-34,36,38,41,43,49,70,71]. Additional socio-demographic data (SES or income groups, education, urban/rural living and relationship status) were reported but not consistently across all studies. Seven studies reported data on participant's income level data, which tended to be higher than average [28,30,31,38,42,49,68]. Sample sizes of the studies ranged from 9 to 1674 participants.

**Table 3:** Characteristics of participants

Study Number, Author and Pub. Year	Mean age, SD or Range	Gender (%Female)	Ethnicity	SES/Income	Education	Quality Metric Score
l. Watson et al, 2005	29.4	100	NS (20% Not Australian born)	96% married, 80% Australian born. Competent at filling in a questionnaire in English	39.2% third level education	5
2. Banks-Wallace et al, 2004	18+	100	African American	NS	NS	4
3. Kolt et al, 2006	74 (SD 6)	66	NS	Urban, Patients from three GP lists. Phone lines at home.	NS	4
4. Nguyen et al, 2002	NS	NS	NS	NS	NS	4
5. Prestwich et al, 2010	23.44	64	NS	Students	Undergraduate	4
6. Rowland et al, 2004	74 (SD 6.2)	69	White (Non-Hispanic) 89%	Income > 35 K US 26%, Married, 57.5%.	Edu. > High school diploma 45%	4
7. Sherman et al, 2006	42.5 (Range 22-64)	100	Caucasian	Rural, 42% Medicare, 43% private insurance, 15% self pay or unknown insurance details, mean BMI 30.6 (78% overweight or obese), 90% with one or more risk factors for CV disease,	NS	4
8. Wilbur et al, 2006	48.6 (Range 40-65)	100	African American	Urban, 60% unmarried, 88% mothers (2.1 children ave.), 70% full time employed, 61% earning > $30 K annually, 57% reporting no 'hardhsips'.	87% some or full third level education	4
9. Baker et al, 2008b	49 (SD 9)	78	NS	NS	NS	3
10. Brownson et al 2005	18+	79.7	95% white	31.3% 35 K+ pa	45% some or full third level Edu.	3
11. Cox et al, 2008	55 (Range 50-70)	100	NS	Urban, English Speakers, married (76%), employed (56.5%), children (2.83). Non-smokers.	Educated (13 years ave.)	3
12. Dinger et al, 2007	41.5 years (Range 25-54 years)	100	86% White	Urban, BMI > 30 (57%), access to email	68% 3rd Level Edu.	3
13. Dubbert et al, 2002	68.7 yrs (60-80 range)	1 (99% Male)	28% Non-white	56.4% rural, 79.6% married/cohabiting, 12.7% tobacco users, 8.8% in financial hardship, 7.4 hrs per week employment, 20% used alcohol, 3.8 co-morbid medical conditions.	51.9% high school or more	3
14. Dubbert et al, 2008	Mean 72 (Range 60 to 85 years)	0 (100% Male)	14% African-American, 86% White	Urban	Majority high school Educated	3
15. Gilson et al, 2008	41.4 (SD 10.4)	91%	NS	All employees at a University	NS	3
16. Jancey et al, 2008	69 (65-74)	67	NS	67% Australian born, Urban ('Metropolitan Perth'), 66% had a partner	NS	3
17. Lamb et al, 2002	50.8 (Range 40-70)	52	NS	NS	NS	3
18. Lee et al, 1997	36.5 (Range 23-54)	100	Latino, African-American, Asian, Pacific Islanders, other (ns)	"Middle class, well educated, English speaking"	"Well educated"	3
19. Matthews et al, 2007	53	100	84% White. 16% African-American/Other	NS	NS	3
20. Merom et al, 2007	49.1 (Range 30-65)	85	NS	Rural and Urban, 74% married, 92.9 English speakers (primarily), 57.7 employed, 72.2% BMI > 25, 90% non-smokers, 81% self rated health good or more.	45.5% university degree	3
21. Ornes and Ransdell, 2007	20 (SD 2.6)	100	"Mostly Caucasian volunteers"	Students	Undergraduate	3
22. Richardson et al, 2007	52 (SD 10.5)	65	76% white, 13% black, 10% other	64% high income > $70,000	NS	3
23. Rosenberg et al, 2009	83 (Range 74-92)	50%	NS	NS	NS	3
24. Whitt-Glover et al, 2008	52 (Range 20-83)	89	Black Americans	Urban, average BMI 34.7, married (49%), 85% had at least one chronic health condition.	96% high school education or higher	3
25. Arbour & Ginis, 2009	48.7 (SD 9.61)	100	90% White	90% Employed	86% Some or full 3rd Level edu.	2
26. Culos-Reed et al, 2008	66 (Range 46-83)	81	96% White	76% retired, 70% higher education, urban	NS	2
27. Currie and Develin, 2001	NS	100	NS	NS	NS	2
28. Darker et al, 2010	40.6 (Range 16-65)	71	NS	NS	NS	2
29. De Cocker et al 2007	48.7 (Range 25-75)	52.8	NS	Urban, 68.1% employed, 63.7% reporting good or better than good health	60% with third level degrees	2
30. Dinger et al, 2005	41.7 (SD 6.8) (Range 25-54)	100	89% White	Employees or spouses of university employees, Overweight or obese (77.7%), not FT students, not pregnant	University degree (69%)	2
31. Engel and Lindner, 2006	62	46	NS	NS	NS	2
32. Foreman et al, 2001	NS	Male and Female	NS	NS	NS	2
33. Humpel et al, 2004	60 (SD 11)	57%	NS	NS	46.9% < 12 yrs edu., 32.1% had a trade edu., 21% Uni.	2
34. Nies et al, 2006	45 (Range 35-60)	100	European-American and African-American	41% > 50 K (US) household income, 49% married, 33% southern American	74% college edu. or higher	2
35. Purath et al, 2004	43.9	100	81.5% White	100% employed at a university (62% in admin/professional), 92% non-smokers, BMI 30.5, 68% married	14.25 years edu. (mean)	2
36. Shaw et al, 2007	40	99	NS	Employed in an urban workplace	NS	2
37. Sidman et al, 2004	43.2	100	NS	NS	NS	2
38. Thomas and Williams, 2006	18-50+	75.5	NS	Employed, Both Urban and Rural locations. 'wide variety of professions, ages, incomes, education standards and levels of health and fitness not considered, disadvantaged in terms of the social determinants of health' 'almost all could be described as sedentary'	NS	2
39. Tudor-Locke et al, 2002	53 (SD 6)	66	NS	NS	NS	2
40. Baker et al, 2008a	40 (SD 8.6)	86	NS	NS	NS	1
41. Hultquist et al, 2005	45 (SD 6 yrs)	100	3 non-white among completers	NS	NS	1
42. Lomabrd et al, 1995	40 (SD 9)	98	NS	University staff	NS	1
43. DNSWH, 2002	(Range 25-65)	NS	NS	Suburban	NS	1
44. Rovniak, 2005	Men (Range 20-44) Women (Range 20-54)	93.5	NS	Urban, at least access to email, sedentary, no more than one health risk factor, BMI < 39.9, no metabolic, pulmonary or CV disease, no bone joint or foot problems, not pregnant.	NS	1
45. Rowley et al, 2007	Children 0-4 Adults not reported	100 (Adults) Children not reported	'There were no children or babies from ethnic minority groups'.	Affluent'	NS	1
46. Talbot et al, 2003	69 (SD 6)	76	17% Non-White	60% > $30 K pa	NS	1
47. Wyatt et al, 2004	NS	NS	NS	NS	NS	1

### Recruitment data reported

Two studies reported all data for all components of recruitment, i.e. where recruitment took place; who conducted the recruitment; the time taken to conduct the planning/preparing and delivery stages [27,51]. Thirty nine studies did report a specified target group (Table 4-Recruitment planning/preparing and implementation). Forty four studies provide some details of where recruitment was conducted [27-49,51-56,58-67,69-73] but the recruitment location was often given vague descriptions, for example "in the community". Most popular were medical/care settings (n = 12) [29-31,33,34,36,38,43,49,51,55,63] or universities (n = 9) [37,40,41,43,44,46,47,62,70]. Other community settings included for example, places of worship [67], hair salons [29], food establishments [29,71] or specific events within such settings, for example meetings for new mothers [51].

**Table 4:** Recruitment planning and implementation (Quality Metric categories)

Study Author (Year)	Where did the Recruitment take place?	Who did the Recruitment?	Time spent Planning/Preparing recruitment	Time spent Executing recruitment (Weeks)	Population Targeted (Yes/No)?	Quality Metric score
l. Watson et al, 2005	Home, health centre visits, at mothers group meetings	Nurses trained in recruitment and research staff	1 month including all training of nurses and intervention by researchers to help with recruitment difficulties.	6 weeks	Yes	5
2. Banks-Wallace et al, 2004	In the community at venues typically used for hosting African American community events	Recruitment Protocol Specialist	NS	21.6 weeks	Yes	4
3. Kolt et al, 2006	By mail and a follow up home visit	Researchers	NS	39 weeks	Yes	4
4. Nguyen et al, 2002	Mainly passively in the community but also used press conferences and info/taster sessions	Public health official	3 years (Rolling development)	NS	Yes	4
5. Prestwich et al, 2010	University	Researchers	NS	2.5 weeks	Yes	4
6. Rowland et al, 2004	Via telephone, direct mail and then at multiple locations and media in the community	Research team members	NS	43.3 weeks	Yes	4
7. Sherman et al, 2006	In a clinic, hair salons-and food establishments	Nurses	NS	0.28 weeks	Yes	4
8. Wilbur et al, 2006	Two federally qualified community health centres serving poor and working class urban populations. Screening and data collection was carried out here to reduce power differences (perceived) and increase trust. Concentrated on an area within a 3-mile radius of the data collection sites. Also interacted in the community at health fairs and presentations.	Team specifically set up to deliver the recruitment made up of AA female nurses, either living in the community or who had family ties to the community.	NS	121.3 weeks	Yes	4
9. Baker et al, 2008b	Local community, GP surgeries, shops, community stalls	NS	NS	21.6 months	Yes	3
10. Brownson et al 2005	Through media, at physicians practices, at community centres, on walking routes, in the community active and passively	Community organisation staff, research staff, physicians	NS	NS	Yes	3
11. Cox et al, 2008	Ads delivered in the community. Screening took place at the community centre	Research assistants	NS	NS	Yes	3
12. Dinger et al, 2007	Local media and electronically	NS	NS	4.3 weeks	Yes	3
13. Dubbert et al, 2002	By mail, phone and at the clinic	Researchers and Research Nurse	NS	NS	Yes	3
14. Dubbert et al, 2008	Primary care medical centre as part of routine care	NS	2 to 3 years	NS	Yes	3
15. Gilson et al, 2008	Via work email	Researchers	NS	NS	Yes	3
16. Jancey et al, 2008	Over the phone to home phone numbers	Researchers	NS	NS	Yes	3
17. Lamb et al, 2002	Via post, phone and info sessions at primary care setting	Researchers, via GP, and staff nurse	NS	NS	Yes	3
18. Lee et al, 1997	Directly and indirectly in the community	Female students trained in recruitment methods	NS	NS	Yes	3
19. Matthews et al, 2007	Clinic	Clinical staff	NS	NS	Yes	3
20. Merom et al, 2007	Passively in the community and actively by phone via another study	Researchers in the NSW Health survey (recruitment by proxy) and researchers on this study	NS	NS	Yes	3
21. Ornes and Ransdell, 2007	University campus	Researchers	NS	NS	Yes	3
22. Richardson et al, 2007	Medical centre	Physicians	NS	NS	Yes	3
23. Rosenberg et al, 2009	Care community	Researchers	NS	NS	Yes	3
24. Arbour & Ginis, 2009	University and Local Community	NS	NS	NS	Yes	2
25. Baker et al, 2008a	University campus	NS	NS	NS	NS	2
26. Culos-Reed et al, 2008	In the community and at the malls	NS	NS	2 weeks	No	2
27. Currie and Develin, 2001	Places where pre and post natal mums engage with health care, shopping and school	NS	NS	NS	Yes	2
28. Darker et al, 2010	In the local media (Passive)	NS	NS	30.3 weeks	No	2
29. De Cocker et al 2007	By mail or phone to participants homes. Indirect but active	NS	NS	NS	Yes	2
30. Dinger et al, 2005	University	NS	NS	NS	Yes	2
31. Engel and Lindner, 2006	In community via newspapers	NS	NS	NS	Yes	2
32. Foreman et al, 2001	NS	Walk leaders and organisers	NS	NS	Yes	2
33. Humpel et al, 2004	Via post. No face to face	NS	NS	NS	Yes	2
34. Nies et al, 2006	Through media and fliers in the community	NS	NS	NS	Yes	2
35. Purath et al, 2004	Health screening day within a university	NS	NS	NS	Yes	2
36. Shaw et al, 2007	Workplace (Health centre)	NS	NS	NS	Yes	2
37. Sidman et al, 2004	Two University campuses	NS	NS	NS	Yes	2
38. Thomas and Williams, 2006	Workplace (Electronically)	NS	NS	NS	Yes	2
39. Tudor-Locke et al, 2002	Diabetes Centre	NS	NS	NS	Yes	2
40. Whitt-Glover et al, 2008	At churches	Church pastors and researchers	NS	NS	Yes	2
41. Hultquist et al, 2005	University	NS	NS	NS	No	1
42. Lomabrd et al, 1995	University campus	NS	NS	NS	No	1
43. DNSWH, 2002	In local area via media and advertising and information	NS	NS	NS	No	1
44. Rovniak, 2005	At multiple locations in the community. Mainly passive.	NS	NS	NS	No	1
45. Rowley et al, 2007	NS	NS	NS	NS	Yes	1
46. Talbot et al, 2003	Senior centres, ads in local newspapers	NS	NS	NS	NS	1
47. Wyatt et al, 2004	NS	NS	NS	NS	Yes	1

Twenty one studies reported who conducted the study recruitment. Most popular recruiters were research staff [28,31,33,34,37,39,51,52,54,62,64,67,72], often with assistance from health professionals like doctors or nurses [29,33,51,65]. Five studies reported using a dedicated "recruitment specialist" [27,30,35,51,69]. Only three studies reported the time spent planning/preparing their recruitment phases [34,51,69]. Eleven studies reported the time spent on implementing recruitment [27-30,32,51,62,63,71,72] and this averaged as 35 weeks, with a range of 2 days to 56 weeks.

### Recruitment procedures and approaches

The reporting of recruitment methods was often sparse and unstructured (Table 5-Number of methods and types of recruitment procedures). Forty five studies provided data on the number of recruitment methods used (mean 2.7, SD 1.97). Sixteen studies relied on one method of recruitment only [33,34,43-45,50-53,56,58,60,62,64,65,72], and 26 studies used between two and four methods [27-32,35-41,54,55,63,66,69-71,73]. We identified two types of recruitment approaches, (i) active approaches; a recruitment method that requires those conducting the study to make the first contact with a participant (e.g. phone calls, face to face invitation, word of mouth, referrals), (ii) passive approaches; a recruitment method that requires a potential participant makes the first contact with the study (e.g. posters, leaflets drops, newspaper advertisements, mail outs). We did not observe any relationship between the quality of recruitment reporting and the number of recruitment strategies used. We did however observe that a number of studies used only passive techniques (n = 21) [32,34,38,41,42,44,46-48,52,54,56,58-62,64,66,67,70], some used a mixture of active and passive techniques (n = 22) [27-31,33,35-37,39,40,49,53,55,57,63,65,68,69,71-73] and a small number used solely active only methods (n = 4) [43,45,50,51].

**Table 5:** Recruitment planning and implementation (Quality Metric categories)

Study Author (Year)	No. Of Methods Used	Procedures including who conducted the recruitment, where it took place and what was done	Active, passive or a mixture of approaches	Quality Metric Score
l. Watson et al, 2005	1	Nurse conducted face to face recruitment at clinics, mothers' group meetings and home visits.	Active	5
2. Banks-Wallace et al, 2004	4	Researchers placed flyers in church bulletins and the community, health practitioner referrals were generated, word of mouth was used and structured pre-intervention meetings took place.	Passive/Active	4
3. Kolt et al, 2006	1	A three phased and sequenced approach was conducted by the researchers, the GP and staff nurse. An invitation letter was sent from the GP surgery a pre-paid response card for those expressing interest. Follow up screening calls then follow up visits to provide info and gain consent.	Passive/Active	4
4. Nguyen et al, 2002	3	A public health official co-ordinated the recruitment and used the local media, network construction and face to face recruitment of volunteer walk leaders. Press conferences and promotional materials were sent to local media outlets, community health centres, libraries, senior's club networks to promote the club. Leaflets on local community settings, ads in free newspapers, promotional messages placed on light panels around the city, community TV ads and features, press releases for local media, newsletters, press conference, celebration events. Comments elsewhere stated that face to face recruitment was the most successful for this study, but this was only used to recruit walk leaders.	Passive/Active	4
5. Prestwich et al, 2010	1	Researchers sent emails to the current students at their university. Course credit or cash were used as an incentive.	Passive	4
6. Rowland et al, 2004	11	Computer assisted telephone interviews (CATI) was initially conducted by researchers. A database of potential participants was screened for telephone numbers. If this was not successful in recruiting the sample size needed the direct mailing was used. Finally, to complete the sample size quota canvassing in the local community (including face to face, door to door, posters and flyers at churches and senior housing units, snowballing, utilising 'community brokers', and newspapers) was conducted. Recruitment was systematic, purposeful and carried out in the order described but was somewhat inequitable as the first screening criterion was the availability of a phone number. It also required significant community assistance to reach those harder to engage.	Active/Passive	4
7. Sherman et al, 2006	2	Active recruitment by a nurse at a health clinic, advertisements in hair salons and food establishments. The paper states that the 'main source of recruitment came from advertisements in the community and word of mouth'.	Active/Passive	4
8. Wilbur et al, 2006	3	Researchers designed a flyer with community input and received advice on where to place it. Emails and newspaper announcements were also used. Recruitment staff distributed print material at specified schools, churches, grocery shops, libraries, clinics, community agencies and community fairs and at 10 presentations in community agencies, clinics, and churches. Email announcement at local medical centre workplaces and an announcement in the community newspaper were used. A good aim of matching the invitation to the invitee and finding the best place to distribute it was a positive here. Unfortunately word of mouth wasn't actively used or reported and only the research team recruitment staff acted as recruiters for face to face recruitment.	Passive/Active	4
9. Baker et al, 2008b	4	Mail drops were carried out and adverts were placed in local papers and posters in GP surgeries and shops. Manned community stalls were also set up. This approach was modified and expanded throughout the recruitment phase as the researchers identified their lack of impact on the target group. However, the methods were mainly passive and not altered to be more engaging or mediating with the target group. It is not specifically stated who conducted the recruitment.	Passive/Active	3
10. Brownson et al 2005	8	Recruitment was initially by proxy during a baseline survey for another piece of work (no details or what survey was). Awareness of the walking group was also promoted at community events, by physician recommendation, trail signage advertising and word or mouth. Recruitment methods were not explicitly reported but intervention communities used participatory approaches to develop their intervention options. Taster events, one off walks, clean up trail days, and 5 media events were held.	Passive/Active	3
11. Cox et al, 2008	1	Research assistants placed advertisements in the local community.	Passive	3
12. Dinger et al, 2007	3	Flyers were placed in the community, emails were sent to university staff and a television advertisement was broadcast. It is not specifically stated who conducted the recruitment.	Passive	3
13. Dubbert et al, 2002	1	A three phased sequenced approach was used. Researchers and a research nurse reviewed medical records. Potential participants were sent a letter and recruited during their scheduled visits with the primary health care providers or following an expression of interest. Nurses conducted a pre screening and financial compensation to offset costs of visits to the centre was provided.	Active/Passive	3
14. Dubbert et al, 2008	1	Participants were recruited via referral by primary care providers, but which specific type of care provider was not reported. It is not specifically stated who conducted the recruitment.	Passive	3
15. Gilson et al, 2008	1	Researchers recruited participants via workplace email.	Passive	3
16. Jancey et al, 2008	1	A two phased sequenced approach was used. Researchers marched electoral roll lists against telephone directory lists to identify potential participants who owned phones. A preceding postcard informing the recruit about the study and the likelihood of a phone call to follow. Phone calls were then made by members of the research team and approximately 9 calls were required to recruit one participant.	Passive/Active	3
17. Lamb et al, 2002	1	A three phased sequenced approach was used. Researchers, assisted by staff nurses sent an eligibility questionnaire to a randomly selected group from a GP client list (GP letters included). This was followed by a letter explaining the study to those expressing an interest and then a phone call to the responders to arrange which info session they could attend.	Passive/Active	3
18. Lee et al, 1997	4	Researchers and trained female students conducted telephone calls, face to face approaches at supermarkets, direct mailing and flyers.	Passive/Active	3
19. Matthews et al, 2007	3	Clinical staff recruited women by letter and phone follow up in two health centres. The paper also states that in another centre clinical populations were recruited, but this is not clearly explained. Women who were also past participants in a case control study and had agreed to take part in future research.	Active/Passive	3
20. Merom et al, 2007	3	Invitation by proxy during the NSW phone Health Survey. Researchers in this study then produced a community based newspaper and sent intranet messages in the area health services (it is not clear what they meant by that).	Passive	3
21. Ornes and Ransdell, 2007	4	Researchers placed newspaper ads and posters on a university campus. Researcher also visited classes on college campus and conducted face to face recruitment on campus.	Passive/Active	3
22. Richardson et al, 2007	3	Researchers placed adverts in a local newspaper and flyers at local hospital, clinics, and other public locations. A listing was placed on a medical research recruitment site. Information and water bottles were given to potential participants and doctors to raise the profile of the study and encourage referrals from doctors.	Passive	3
23. Rosenberg et al, 2009	2	Researchers used flyers and information meetings.	Passive/Active	3
24. Whitt-Glover et al, 2008	5	Pastors who attended luncheons regarding health promotion and disease prevention strategies among African Americans were recruited to help introduce the intervention and aid recruitment of participants. Following this, researchers placed flyers in churches, bulletins in newsletters, announcements at Sunday services and held information meetings.	Active/Passive	3
25. Arbour & Ginis, 2009	2	Posters and internet ads were sent as part of an employee health programme. It is not specifically stated who conducted the recruitment.	Passive	2
26. Culos-Reed et al, 2008	4	Posters, cards on food hall tables and two community newspapers were used to circulate information. Three presentations were held at local health programme meetings. It is not stated who conducted the recruitment.	Passive/Active	2
27. Currie and Develin, 2001	4	Flyers were placed at the local maternity wards, doctors' surgeries, early childhood centres, day care centres, immunization clinics, baby product stores and playgrounds. Adverts placed in school bulletins; local newspapers and also paid adverts in newspapers. Information sessions were conducted for new mothers in childhood centres. It is not specifically stated who conducted the recruitment.	Passive/Active	2
28. Darker et al, 2010	2	Adverts were placed in local newspapers. Radio interviews were conducted. It is not specifically stated who conducted the recruitment.	Passive	2
29. De Cocker et al 2007	3	Telephone calls and postal mail invites to 2500 randomly selected members of the registered population. A multi-media campaign was carried out to raise awareness of the programme. It is not specifically stated who conducted the recruitment.	Active/Passive	2
30. Dinger et al, 2005	2	Emails were sent to university staff and adverts were placed on the University television station. It is not specifically stated who conducted the recruitment.	Passive	2
31. Engel and Lindner, 2006	1	A 'local Media campaign' was conducted. It is not specifically stated who conducted the recruitment.	Passive	2
32. Foreman et al, 2001	2	This qualitative paper did not clearly describe the processes behind their recruitment approach. It emphasises the need for the walk leaders and organisers to become actively engaged in the process and how interpersonal approaches are highly necessary and more effective in engaging a broader range of participants or specific target groups.	Active/Passive	2
33. Humpel et al, 2004	1	Letters were sent to individuals listed in an insurance company client list, with follow up letters to non-responders. It is not specifically stated who conducted the recruitment.	Passive	2
34. Nies et al, 2006	2	Flyers were placed in the local community and the programme was promoted on the radio. It is not specifically stated who conducted the recruitment.	Passive	2
35. Purath et al, 2004	1	Participants were recruited at annual workplace health screenings. May have been pre-notified but this isn't stated. It is not specifically stated who conducted the recruitment.	Active	2
36. Shaw et al, 2007	4	The study was promoted via workplace intranet, staff newsletter and flyers. Emails were sent to managers of departments to be forwarded to staff. It is not specifically stated who conducted the recruitment.	Passive	2
37. Sidman et al, 2004	1	Flyers were posted on two University campuses. It is not specifically stated who conducted the recruitment.	Passive	2
38. Thomas and Williams, 2006	1	Emails were distributed in the workplace. It is not specifically stated who conducted the recruitment.	Passive	2
39. Tudor-Locke et al, 2002	1	Recruited at/after an diabetes education session. Convenience sample, first come first serve. It is not specifically stated who conducted the recruitment.	Active	2
40. Baker et al, 2008a	3	Posters and newsletters were placed on a University campus. Emails were sent to University staff. It is not specifically stated who conducted the recruitment.	Passive	1
41. Hultquist et al, 2005	2	Flyers were placed on a University campus and in the surrounding area. The study was publicised in a local newsletter. It is not specifically stated who conducted the recruitment.	Passive	1
42. Lomabrd et al, 1995	2	Newspaper advertisements and flyers were posted on campus at a University. It is not specifically stated who conducted the recruitment.	Passive	1
43. DNSWH, 2002	4	Flyers distributed via letter box drop. Use of a 'feature' newspaper article. Information sent to local community groups (e.g. Rotary and Lions), schools, preschools, playgroups, community nurses, doctors' surgeries, local rugby club, and local business (e.g. chemists' shops, real estate agents, car dealerships). Poster and flyers placed in parks, at bus stops, local streets, shops, libraries and other public facilities. It is not specifically stated who conducted the recruitment.	Passive	1
44. Rovniak, 2005	5	The methods are reported as: the use of local list-servs for direct mailing; churches; a news brief on a local radio and television station, a university newspaper article, and flyers. It is not specifically stated who conducted the recruitment.	Passive	1
45. Rowley et al, 2007	Unclear	The paper reports only the following details regarding recruitment: 'There was an enthusiastic response from invited mothers and many requests to join from other who had heard about the programme through local publicity and word of mouth'. It is not specifically stated who conducted the recruitment.	Passive/Active	1
46. Talbot et al, 2003	2	Participants were recruited through senior centres and advertisements in local newspapers. It is not specifically stated who conducted the recruitment.	Passive/Active	1
47. Wyatt et al, 2004	1	Word of mouth at a 'kick start' session. It is not specifically stated who conducted the recruitment.	Active	1

**Figure 2:**
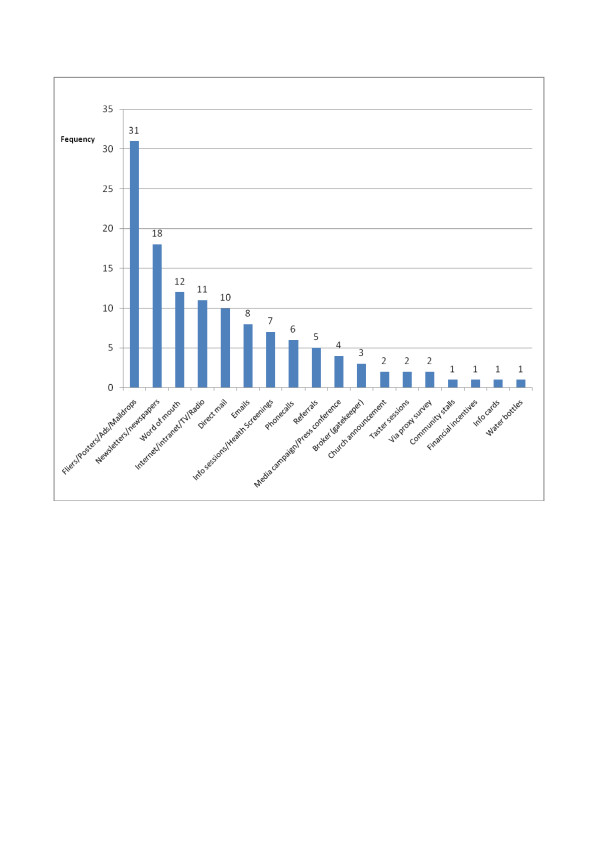
**Methods of recruitment and frequency of use from all included studies (n = 47)**.

Passive recruitment methods, which require no interaction with the potential participants, were popular (Figure 2). Flyers/posters/advertisements/mail drops were the most cited approach used, appearing in 31 studies. This was almost twice as prevalent as the second most popular approach, newsletters/newspaper articles (n = 18) and was nearly three times more frequently used than word of mouth. Word of mouth appeared in 12 studies, but we were unable to identify whether this was a proactive recruitment strategy or a reactive strategy, responding to low recruitment numbers. Less popular methods included medical and health insurance referral, invitations derived from clinical or employment data, study information sessions, resident listings, announcements at group meetings or community events and information stands.

### Locations for recruitment, interventions and target populations

Table 6 presents data on the setting and location of recruitment and the study. We observed some studies that "matched" where the recruitment was conducted with where the intervention was delivered. Culos-Reed et al, 2008 reported recruiting participants for a mall walking study at the mall where the intervention was going to be delivered [71]. Other studies did not match in this way, and recruited in many different locations, often relying on print material alone, and requiring potential participants to attend a location which may not be easily accessible to them. Studies reported that they were "community-based" (n = 25) [27-31,35,36,42,48-52,54-58,61,63,68,69,71-73] but asked community members to travel into a research setting to begin the process of participation; for example medical centres or universities (n = 20) [29,30,33-38,41,43,46,47,49,56,62-67]. These interventions used a mixture of recruitment approaches including media events and led walking groups, face to face interventions (e.g. counselling, pedometers) or mediated interventions, such as internet, e-health and mobile phone technology [74].

**Table 6:** Settings and Locations of recruitment, study and populations

Study Author (Year)	Stated Study setting	Target population	Where did the Recruitment take place?	Intervention delivery site	Where Participants came from	Quality Metric Score
l. Watson et al, 2005	Community	Post-natal mothers	Home, health centre visits, at mothers group meetings	Community via lead walks	Mothers using community health centres or early childhood health centres or mothers visited by local childcare nurses	5
2. Banks-Wallace et al, 2004	Community Setting: African American (AA)	African American women in a local community (Minority group)	In the community at venues typically used for hosting African American community events	Local community venue used for hosting AA community events	African American Community	4
3. Kolt et al, 2006	Community	Older sedentary adults (> 65)	By mail and a follow up home visit	By phone and a home visit at screening (Community)	GP Patient lists	4
4. Nguyen et al, 2002	Community	General community	Mainly passively in the community but also used press conferences and info/taster sessions		Community	4
5. Prestwich et al, 2010	University	University students	University	University	University students	4
6. Rowland et al, 2004	Community	Sedentary older adults	Via telephone, direct mail and then at multiple locations and media in the community	At home	Community members identified through a commercial database of household data	4
7. Sherman et al, 2006	Community (Rural)	Rural women	In a clinic, hair salons-and food establishments	Clinical centre	Residents in the local community	4
8. Wilbur et al, 2006	Community and Home	African American Women	Two federally qualified community health centres serving poor and working class urban populations. Screening and data collection was carried out here to reduce power differences (perceived) and increase trust. Concentrated on an area within a 3-mile radius of the data collection sites. Also interacted in the community at health fairs and presentations.	Community health centres. Purposely chosen to reduce power differences and increase trust. Within three miles of the participants residential area	Predominantly African American women within a 3-mile radius of the intervention centre	4
9. Baker et al, 2008b	Community	Community members in areas of high deprivation (> 15% SIMD)	Local community, GP surgeries, shops, community stalls	University campus	Residents within a surrounding area of West Glasgow university (1.5 km)-defined as a suitable walking distance from intervention site	3
10. Brownson et al 2005	Community (Rural USA)	Rural community members	Through media, at physicians practices, at community centres, on walking routes, in the community active and passively	Community	Within targeted community	3
11. Cox et al, 2008	Community	Previously sedentary older women	Ads delivered in the community. Screening took place at the community centre	Community centre	Recruited from the community'	3
12. Dinger et al, 2007	University	Insufficiently active women (University staff and local community members)	Local media and electronically	Intervention delivered by email (Virtual)	University staff and local community	3
13. Dubbert et al, 2002	Care setting (Veterans Affairs Medical Centre)	Elderly primary care patients	By mail, phone and at the clinic	Medical centre	Attendees at a Veterans Affairs Medical centre	3
14. Dubbert et al, 2008	Care setting	Elderly veterans	Primary care medical centre as part of routine care	Primary care clinic	Primary care clinics for veterans	3
15. Gilson et al, 2008	Workplace (University)	Work-place employees	Via work email	University	University employees	3
16. Jancey et al, 2008	Community	Older adults	Over the phone to home phone numbers	Selected green space areas within the neighbourhood local to the recruited participants	Urban areas of Perth, identified through electoral roll	3
17. Lamb et al, 2002	Care (Primary care)	Middle aged adults	Via post, phone and info sessions at primary care setting	Primary care facilities	Primary care client list	3
18. Lee et al, 1997	Community	Sedentary ethnic minority women	Directly and indirectly in the community	Baseline screening at a University, then indirectly delivered to participants homes	Members of women, children and infant groups, local area San Diego	3
19. Matthews et al, 2007	Care: Clinical and Home (Community) setting	Breast cancer survivors	Clinic	Clinical centres	Former or existing clinical populations	3
20. Merom et al, 2007	Community	Inactive adults	Passively in the community and actively by phone via another study	This was a passively delivered intervention and participants received intervention material and equipment entirely by post.	Non-clinical sample of individuals in the community	3
21. Ornes and Ransdell, 2007	University	Women	University campus	University	University	3
22. Richardson et al, 2007	Care: Clinical	Adults with type 2 diabetes	Medical centre	Clinical centre	Adults with diabetes living in the community	3
23. Rosenberg et al, 2009	Care setting (Retirement community)	Older adults	Care community	Continuing care retirement community	Residential care facility	3
24. Whitt-Glover et al, 2008	Churches	Black adult, church attendees	University and Local Community	Church meeting rooms	Church groups	3
25. Arbour & Ginis, 2009	Workplace	Women in the workplace	University campus	Workplace (University)	University	2
26. Culos-Reed et al, 2008	Community: Malls	NS	In the community and at the malls	Mall	Mall users from the local community	2
27. Currie and Develin, 2001	Community	Mothers and young children	Places where pre and post natal mums engage with health care, shopping and school	Community	NS	2
28. Darker et al, 2010	Clinical lab setting	NS	In the local media (Passive)	Laboratory	NS	2
29. De Cocker et al 2007	Community	'General population' adults in a local community	By mail or phone to participants homes. Indirect but active	In the community with contact via phone and mail for pedometer packs	General population members as listed on the population register	2
30. Dinger et al, 2005	University	Female employees or spouses of university employees	University	University campus	University staff and spouses	2
31. Engel and Lindner, 2006	Community	Adults with type 2 diabetes	In community via newspapers	At research institute or at home	Local Community	2
32. Foreman et al, 2001	Community	Community members	NS	NS	NS	2
33. Humpel et al, 2004	Community	Over 40 year old community members	Via post. No face to face	No face to face contact, but participants encouraged to walk in their local area	Insurance company client list	2
34. Nies et al, 2006	Community	European American and African America women.	Through media and fliers in the community	NS	NS	2
35. Purath et al, 2004	Workplace (University)	Women in the workplace	Health screening day within a university	University	Staff attending a voluntary university provided health screening as part of a wellness programme	2
36. Shaw et al, 2007	Workplace (Health Centre staff)	Men and women in the workplace	Workplace (Health centre)	Workplace (Urban workplace)	Health Centre staff	2
37. Sidman et al, 2004	University (Seems Uni)	Sedentary women	Two University campuses	NS	NS (Recruited on Uni campus)	2
38. Thomas and Williams, 2006	Workplace	Workplace staff (Excluding hospital and community services staff)	Workplace (Electronically)	NS	Workplace staff (Dept. of Human Services staff)	2
39. Tudor-Locke et al, 2002	Health centre	Sedentary diabetes sufferers	Diabetes Centre	Diabetes care centre	Diabetes care centre	2
40. Baker et al, 2008a	University	NS	At churches	University campus	University campus	1
41. Hultquist et al, 2005	University	NS	University	University	University campus	1
42. Lomabrd et al, 1995	University	NS	University campus	University	University staff	1
43. DNSWH, 2002	Community	NS	In local area via media and advertising and information	Community	Residents of local community	1
44. Rovniak, 2005	Community	NS	At multiple locations in the community. Mainly passive.	NS	NS (Seems community)	1
45. Rowley et al, 2007	Community	Parents and children	NS	In the community along planned walking routes in and out of parks/green spaces	Affluent community in semi-rural England'	1
46. Talbot et al, 2003	Community (Home)	Older adults	Senior centres, ads in local newspapers	University clinic	Local Community	1
47. Wyatt et al, 2004	Community	State wide residents of the community	NS	Worksite and Church via a starter kit	Workplaces and church	1

### Recruitment rates and efficiencies

We originally planned to calculate recruitment rates and efficiency ratios for each study but we were unable to do so due to missing data (Table 7-Recruitment rates and efficiency ratios). Only three studies provided all the data points [33,36,65]. We were able to calculate a weekly recruitment rate using the final numbers of participants divided by the time spent recruiting in weeks for eleven studies (mean 38 participants per week, range 1 to 268 participants per week). We were not able to see any pattern between recruitment approaches and weekly rates. Two studies reported some data on the efforts needed to undertake recruitment. Jancey et al (2008) reported that after potential participants had received invitation cards it took approximately 9 calls to recruit one participant [53].

**Table 7:** Recruitment rates and efficiency ratios

Study Author (Year)	Pool	Invited	Responded	Started	Efficiency A (%) (Started/Pool)	Efficiency B (%) (Started/Invited)	Efficiency C (%) (Started/Responded)	Efficiency D (N) (Started only)	Weekly Recruitment Rate	Quality Metric Score
l. Watson et al, 2005	NS	NS	NS	139	-	-		139.0	23.17	5
2. Banks-Wallace et al, 2004	NS	NS	38	21	-	-	55.3	21.0	0.97	4
3. Kolt et al, 2006	NS	NS	NS	186	-	-	-	186.0	4.77	4
4. Nguyen et al, 2002	NS	NS	NS	NS	-	-	-	NS		4
5. Prestwich et al, 2010	NS	NS	173	149	-	-	86.1	149.0	59.60	4
6. Rowland et al, 2004	73828	NS	NS	582	0.8	-	-	582.0	13.44	4
7. Sherman et al, 2006	1700	NS	75	75	4.4	-	100.0	75.0	267.86	4
8. Wilbur et al, 2006	NS	NS	NS	281	-	-	-	281.0	2.32	4
9. Baker et al, 2008b	NS	NS	169	80	-	-	47.3	80.0	3.70	3
10. Brownson et al 2005	NS	NS	NS	NS	-	-	-	-		3
11. Cox et al, 2008	NS	NS	1312	124	-	-	9.5	124.0		3
12. Dinger et al, 2007	NS	NS	87	74	-	-	85.1	74.0	17.21	3
13. Dubbert et al, 2002	576	576	253	212	36.8	36.8	83.8	212.0		3
14. Dubbert et al, 2008	572	572	NS	224	39.2	39.2	-	224.0		3
15. Gilson et al, 2008	NS	NS	102	70	-	-	68.6	70.0		3
16. Jancey et al, 2008	NS	7378	NS	260	-	3.5	-	260.0		3
17. Lamb et al, 2002	26500	2000	960	260	1.0	13.0	27.1	260.0		3
18. Lee et al, 1997	NS	NS	387	128	-	-	33.1	128.0		3
19. Matthews et al, 2007	117	117	102	36	30.8	30.8	35.3	36.0		3
20. Merom et al, 2007	NS	NS	692	369	-	-	53.3	369.0		3
21. Ornes and Ransdell, 2007	NS	NS	210	121	-	-	57.6	121.0		3
22. Richardson et al, 2007	NS	NS	76	35	-	-	46.1	35.0		3
23. Rosenberg et al, 2009	400	400	NS	22	5.5	5.5	-	22.0		3
24. Whitt-Glover et al, 2008	NS	NS	NS	87	-	-	-	87.0		3
25. Arbour & Ginis, 2009	NS	NS	129	75	-	-	58.1	75.0		2
26. Culos-Reed et al, 2008	NS	NS	87	52	-	-	59.8	52.0	26.00	2
27. Currie and Develin, 2001	NS	NS	110	NS	-	-	-	NS		2
28. Darker et al, 2010	NS	NS	176	132	-	-	75.0	132.0	4.36	2
29. De Cocker et al 2007	5000	4065	NS	1674	33.5	41.2	-	1674.0		2
30. Dinger et al, 2005	NS	NS	43	36	-	-	83.7	36.0		2
31. Engel and Lindner, 2006	NS	NS	NS	57	-	-	-	57.0		2
32. Foreman et al, 2001	NS	NS	NS	NS	-	-	-	NS		2
33. Humpel et al, 2004	NS	982	429	399	-	40.6	93.0	399.0		2
34. Nies et al, 2006	NS	NS	313	253	-	-	80.8	253.0		2
35. Purath et al, 2004	NS	NS	NS	287	-	-	-	287.0		2
36. Shaw et al, 2007	NS	NS	NS	35	-	-	-	35.0		2
37. Sidman et al, 2004	NS	NS	NS	114	-	-	-	114.0		2
38. Thomas and Williams, 2006	3500	NS	1195	1195	34.1	-	100.0	1195.0		2
39. Tudor-Locke et al, 2002	NS	9	9	9	-	100.0	100.0	9.0		2
40. Baker et al, 2008a	NS	NS	61	52	-	-	85.2	52.0		1
41. Hultquist et al, 2005	NS	NS	73	58	-	-	79.5	58.0		1
42. Lomabrd et al, 1995	5000	NS	NS	135	2.7	-	-	135.0		1
43. DNSWH, 2002	NS	NS	NS	NS	-	-	-	NS		1
44. Rovniak, 2005	NS	NS	235	65	-	-	27.7	65.0		1
45. Rowley et al, 2007	NS	NS	NS	165	-	-	-	165.0		1
46. Talbot et al, 2003	NS	NS	64	40	-	-	62.5	40.0		1
47. Wyatt et al, 2004	NS	NS	735	735	-	-	100.0	735.0		1

### Developing Recruitment Approaches

We identified factors that may have helped or hindered recruitment from qualitative [57,64,69] and protocol [27,28,30,35] papers. These factors emerged as possible principles of recruitment and were related to training, engaging possible participants in the recruitment process and allowing sufficient time to pilot-test approaches. Watson et al (2009) used trained post-natal health care staff to actively recruit participants during their first home and health centres visits, and at group meetings for new mothers [51]. Recruitment approaches used by Banks-Wallace et al (2004) were based on a 5 month needs assessment study of the concerns and priorities of their target group [27]. The authors reported this process established trust between the research team and participants and ensured active participation in the study and in fact over-recruited from this population. Nguyen et al (2002) reported promoting participation via word of mouth, e.g. one participant tells/recruits another participant [69]. These appeared to have more impact on recruitment than passive approaches like posters or media stories [69]. These data suggest that developing recruitment approaches is a time and resource intensive activity, requiring skilled research and recruitment staff.

## Discussion

We conducted a systematic review to examine the reported recruitment procedures of walking studies, in order to identify the characteristics of effective recruitment and the impact and differential effects of recruitment strategies among particular population groups. We identified the need for a common understanding of the recruitment process for walking studies in terms of conceptual definition, defining effectiveness and more detailed reporting. Due to the heterogeneity of studies we were not able to identify what specific recruitment approaches were most successful with particular population groups.

We identified eighteen recruitment strategies from 47 studies but did not see any relationship between one particular strategy or group of strategies and recruitment rates. Many studies blended different recruitment approaches and strategies, adopting an almost "trial and error" approach. Only two studies reported the effectiveness of their approaches to recruitment [28,35]. We were able to distinguish active and passive recruitment approaches. Further research is needed to directly compare specific recruitment strategies.

Very few studies examined the successes of recruitment approaches to physical activity interventions. Harris et al (2008) conducted a randomized controlled trial of four recruitment strategies in their physical activity promotion intervention study for older adults [75]. The authors reported that telephone follow up a week post invitation significantly increased recruitment compared to invitation only. Certainly the principle of follow up was found in a number of our included studies [53,63,72] but we could not assess the efficacy of these strategies. The efficacy of phone recruitment has been questioned by Margitic et al (1999) [76] who compared three recruitment strategies for Project ACT: patient mailings, office-based questionnaires and telephone contact. However their participants were not randomized to a particular strategy. The authors also reported that despite telephone recruitment appearing to be productive this strategy was dropped in two out of eight recruitment sites on cost grounds. This behavioural approach of using phone follow up has previously been reported to be more effective than no follow up in changing physical activity and walking behaviour [10,23] and certainly warrants further testing in terms of a possible recruitment strategy.

Tai and Iliffe's (2000) experiences of conducting physical activity studies also support our observation that piloting and pre-testing of recruitment methods would improve rates of recruitment and precision in recruiting specific target groups [77]. Our review clearly shows that current recruitment strategies resulted in recruiting mostly white, well-educated, middle aged women. The attraction of walking projects to particular social groups has also been reported in previous evaluation studies of community walking programmes both in the UK [78] and USA [27,31]. Our review found that recruitment rates were poorer for men, especially within workplace or community settings but we were unable to determine if it is a fault of the recruitment, or the offer of walking or a combination of both that is at fault.

We identified a number of studies that "matched" where the recruitment was conducted, with where the intervention was delivered. This principle supports the notion that connecting the place of recruitment and intervention may be more efficient both for the participants, recruiters and interventions teams. We found studies that did not effectively match these aspects and perhaps this was reflected in the total number of participants recruited and the longer time it took to conduct their recruitment phase. For example, Baker et al (2008) reported that participants were expected to travel to the university to receive their intervention. We found little data on the time spent planning/preparing and implementing recruitment so any potential learning from recruitment remains unreported [63].

We identified a number of studies that also aimed to match those recruiting with those being recruited. Banks-Wallace et al (2004) reported in detail their use of a recruitment mediator [27]. The mediator was the same gender as the target group, was a prominent local figure, trained in delivering community interventions and female. Her role was to introduce the study to key significant figures in the area and increase awareness, to assist directly with the recruitment phase and to introduce the researchers to the potential participants at an information session. Banks-Wallace et al (2004) described this approach as increasing trust and decreasing differences between the recruiters and recruited [27].

**Figure 3:**
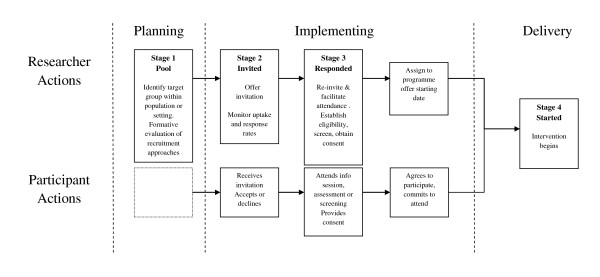
**Conceptual framework for the stages and steps of recruitment with actions for researchers and participants**.

Our review clearly found there was very little consistency in the definition or reporting of recruitment. We found many different interpretations of (i) what is the recruitment process? and (ii) what is an appropriate metric for evaluating the effectiveness of recruitment? The lack of conceptual clarity about recruitment as a process is surprising and potentially impacts on cost-effectiveness. The RE-AIM framework emphasises the need to judge the success of an intervention from both the reach and uptake of an intervention [79]. In light of this we constructed a conceptual framework for our review by defining the stages of recruitment and potential pool of participants (Figure 3). This framework offers a starting point for further debate and refinement. The framework offers a clear concept of the stages and steps of recruitment and the chance to record the numbers of participants at each stage and action.

Our framework divided recruitment into two phases, planning/preparing and implementation, with four stages involving discrete actions by researchers/recruiters, (i) identification of participant pools, (ii) invitation and monitor response and uptake, (iii) assessment, screening and facilitation and (iv) re-invitation of responders, before the delivery of intervention to starters. This framework highlights the actions needed at the start of a recruitment process, i.e. planning/preparing the recruitment process. It also emphasises the importance of the reach of an intervention i.e. the pool of participants used to provide recruits. This differs from the recent new CONSORT framework which asks for dates of recruitment period (i.e. delivery) and enrolment stage [18]. CONSORT stipulates data must be reported for numbers of participants eligible for study which we feel not only ignores the overall pool of possible participants, especially in community based studies of walking interventions, but also ignores the population deemed ineligible, as seen in pre-screenings of patient lists for existing conditions [18]. The "pool" of participants perhaps provides a more realistic denominator for assessing overall recruitment rates. This metric will allow new studies to (i) consider if the recruitment was efficient (i.e. study recruited expected numbers of participants) and/or (ii) consider if it was effective (i.e. study recruited the right target group), and/or (iii) reflect the true costs of all recruitment actions within overall cost benefit calculations. The need for better reporting of recruitment actions and numbers is essential to improve the assessment of present recruitment strategies. This view is mirrored in recruitment studies of other health behaviours, and better reporting must begin before we can start to identify which strategies provide the best recruitment rates [80].

The results of our review were limited to walking intervention studies. We were limited by only including studies written in the English language. We were limited by what was reported in papers but our consistent application of inclusion, quality and data extraction criteria have illustrated the need for improvement in both the reporting and science of recruitment. As journals look to keep research reports within word limits, it is likely that there will continue to be a lack of journal space to report recruitment details, and we would like to call on editors and authors to report recruitment details or provide short methods papers for the insight of future researchers. As far as we are aware this review is the first of its kind focusing on one domain of physical activity behaviour. The lack of understanding and studies into recruitment may reflect some of the findings about the existing weaknesses of the evidence base for walking interventions, e.g. lack of generalisability of interventions across different social groups [10].

The evidence base for the benefits of walking is now expanding but until it is clearer what strategies are effective in both recruiting and initiating people to begin walking, such benefits may be out of reach for particular population groups. Practitioners would benefit from the assurance of having an evidence based best practice model which details how best to recruit participants as well as what is the best intervention to promote walking. Our conceptual framework offers researchers, practitioners and policy makers a way forward to develop and assess the success of a recruitment strategy to target particular groups. The model offers options through the four stages to assess how many people are responding and engaging in a walking intervention, but also whether any bias is occurring and if efforts need to be refined towards a specific group. It could also provide a true picture of the costs of the intervention as the inclusion of recruitment development and implementation should be included in economic evaluations.

The results of our review could translate into a series of recruitment principles for further evaluation by researchers. These principles include (i) form recruitment plans and strategies on evidence of what the target group feels would be appropriate, based on formative research, (ii) conduct a pilot phase of testing, (iii) recruit in places where the participants are located, (iv) allow sufficient time to recruit participants and monitor the uptake, (v) provide training in recruitment methods for recruitment staff, (vi) monitor the participants response to recruitment approaches and use different recruitment strategies where necessary.

The future of walking and physical activity promotion will lie not only in establishing the effectiveness of different interventions but also in improved recruitment practice. Currently, generalisability is limited by reach within studies; but while the current methods being used are applied, the current limited reach will prevail. We offer principles for recruitment that require further evaluation, (i.e. matching "where to where" and "who to who"). Future research to identify "what is effective recruitment?" may best lie in identifying approaches that reflect the needs and expectations of hard to recruit target groups. This will allow researchers the opportunity to investigate the strategic use of the *right recruitment methods*, for the *right group*, in the *right order*.

## Competing interests

The authors declare that they have no competing interests.

## Authors' contributions

CF, GB & NM conceived of the study, and participated in its design, coordination and helped to draft the manuscript. CFtz & CMcA participated in its design and coordination and helped to draft the manuscript. CF, GB & AM participated in review screening, data extraction and helped to draft the manuscript. All authors read and approved the final manuscript.
